# Editorial: The role of the cerebellum in dementia and neurodegenerative diseases

**DOI:** 10.3389/fnins.2023.1229624

**Published:** 2023-06-16

**Authors:** Sofia Toniolo, Iolanda Pisotta, Mario Manto

**Affiliations:** ^1^Nuffield Department of Clinical Neurosciences, John Radcliffe Hospital, University Oxford, Oxford, United Kingdom; ^2^NeuroRobot Lab, Laboratorio di Neuroriabilitazione Robotica, IRCCS Fondazione Santa Lucia, Rome, Italy; ^3^Department of Neurosciences, University of Mons, Mons, Belgium; ^4^Service de Neurologie, CHU-Charleroi, Charleroi, Belgium

**Keywords:** cerebellum, CCAS, cognition, dementia, neurodegeneration

The neuroscience of the cerebellum has been modified substantially in these last 40 years. The longstanding idea that the cerebral cortex is the sole neural correlate of human cognition is no more valid (Magielse et al., 2022). In particular, the exceptional volumetric increase of the lateral cerebellum in conjunction with its connectivity with the cerebral cortical system has been linked to non-motor functions and mental operation in primates. Lateral cerebellar lobules crura I-II and their reciprocal connections to the cerebral cortical association areas have substantially expanded in great apes and humans (Magielse et al., 2022). Along with the increase in the ventral portions of the dentate nucleus and the spreading prefrontal-cerebellar connectivity, this suggests that modular cerebellar adaptations support cognitive functions in humans. Accordingly, the role of the human cerebellum in high-order cognitive function has gained increasing attention in recent years. Since the original description of the “Cerebellar Cognitive Affective Syndrome (CCAS)” (Schmahmann's syndrome) by Schmahmann and Sherman (1998), the field has gained a deeper understanding of the mechanisms that link functional or structural disruption of selective cerebellar areas to changes in cognitive function. Evidence has now accumulated across diseases that primarily affect the cerebellum (Koziol et al., 2014), as well as neurodegenerative diseases where the cerebellum has been traditionally seen as a “silent bystander” (Jacobs et al., 2017). This Research Topic explored how changes in the interplay between cerebellum, cortex and other relay brain structures such as the basal ganglia contribute to cognitive performance, and how vascular lesions in specific cerebellar structures map onto distinct cognitive domains.

Marvel et al. used quantitative susceptibility mapping (QSM) to measure susceptibility mass (an index of iron content) and quantify structural atrophy in the cerebellar dentate and basal ganglia in patients with SCA3 (*n* = 10), SCA6 (*n* = 6) and nine healthy controls. They found that in SCA6 patients, a lower iron content in the dentate nucleus and higher in the basal ganglia was associated with lower motor and cognitive performance (in the language domain of the Montreal Cognitive Assessment and at a finger tapping test), while this association was not found in patients with SCA3. Conversely, lower basal ganglia volume was the driver of worse cognitive scores in patients with SCA3. This suggests that different mechanisms underly a decline in cognition across different SCA subtypes and supports the idea that iron relocation from the dentate to the basal ganglia is associated with worse outcomes in SCA6.

The study by Xu et al. investigated patterns of resting state fluctuations at fMRI across three frequency bands, e.g., conventional (0.01–0.08 Hz), slow-4 (0.027– 0.073 Hz), and slow-5 (0.01–0.027 Hz), in a cohort of patients with Parkinson's disease (PD) with (*n* = 28) and without apathy (*n* = 19) and healthy controls (*n* = 32). Patients with PD showed lower regional homogeneity (ReHO), a measure of local functional synchronization of spontaneous neuronal activity, across all three frequency bands compared to healthy controls in the cerebellum, regardless of the presence of apathy. Conversely, other metrics such as the amplitude of low-frequency fluctuation (ALFF) in the right anterior cingulate and middle frontal gyrus were able to distinguish between PD with and without apathy. Decreased ReHo in the Crus II was observed in the conventional and slow-4 frequency bands in PD patients compared to controls, while this was observed in the slow-5 frequency band in lobules VII and VIII of the cerebellum. Given the importance of basal ganglia dysfunction in patients with PD, this study reinforces the idea that the physiological relationship between cerebellum and basal ganglia might be altered across different neurodegenerative diseases. It also contributes to the growing literature showing that these cerebellar structures are important for cognition not only in healthy aging and patients with a primary dementia such as Alzheimer's Disease (AD) (Gellersen et al., 2017), but also in patients with PD (Gardoni et al., 2023).

Acharya et al. investigated functional connectivity changes between cerebellum, basal ganglia and the cortex in 40 patients with subcortical vascular mild cognitive impairment (svMCI) and 40 healthy controls, using a seed-based approach with the caudate as region of interest within the basal ganglia. Results showed that the amount of cerebellar atrophy within the default mode, salience, and frontoparietal networks correlated with their counterpart in the cerebral cortex. SvMCI showed atrophy in several areas of cerebellum, including the lobule VI, IX, and X and Crus I, compared to healthy controls, as well as reduced functional connectivity between the caudate and lobule IV, VI, Crus I and II, VIIIa, VIIb, X, which are located within default mode, salience and frontoparietal cerebellar networks. Gray matter atrophy in default mode, salience, fronto-parietal and dorsal attention networks correlated with scores at the mini mental state examination and immediate recall of the Auditory Verbal Learning Test (AVLT), while disrupted functional connectivity between the caudate and the frontoparietal and default mode networks in the cerebellum was associated with scores at delayed recall of the AVLT. These regions are considered key cognitive areas of the cerebellum (Buckner et al., 2011) and have found to be altered along the disease course in patients with MCI due to AD and AD dementia (Toniolo et al., 2018, 2020). This study extends this finding to MCI of vascular etiology and links these functional and anatomical changes to the cognitive impairment experienced by these patients.

Whilst the previous study focused on the cumulative impact of multiple small vascular lesions across the brain in determining increased atrophy, reduced functional connectivity and worse cognitive performance, Liu et al. looked at large-scale ischemic lesions and mapped lesion localization to the cognitive domain involved in 45 patients and 30 healthy controls. Patients had lower overall global cognition, measured by the Addenbrooke's Cognitive Examination. Moreover, attention and executive functions, indexed by tests including Trail Making, Digit Span and Stroop, were the most affected single cognitive domains. Patients with lesions in the right cerebellar hemisphere had worse executive functions compared to their left counterpart but not worse global cognition. Lesions in lobules VI, VIII, and IX were associated with lower global cognitive performance, with the highest association found for lobule VI (64.7% patients exhibiting impaired performance). Preferential associations between lesions within lobule VI, Crus I and lobule IX were specifically associated respectively with worse attention and working memory (Lobule VI), executive functions (Crus I), and episodic memory (lobule IX).

Overall, data gathered through these studies highlight the importance of cerebellar-basal-ganglia-cortical circuitries in determining cognitive performance. This has been found across different modalities (QSM, resting state fMRI, structural MRI), and across different diseases (SCA3 and 6, PD, patients with ischemic strokes). Whilst the association between lesion localization and cognitive domain affected might rely on the specific population examined, a prominent involvement of attention, executive function and language, as well as global cognition, is in line with the “dysmetria of thought” hypothesis, which is further corroborated by the disruption seen within fronto-parietal, salience and default mode networks. Since the seminal works of Larsell on the anatomy of the cerebellar lobules, our appraisal of the cerebello-cerebral connectivity has revealed that a functional disconnection occurs in neurological and psychiatric conditions such as Alzheimer's disease and major depressive disorder, causing a dysmetria of thought (Haines, 2023; Leggio, 2023). As pointed out by Schmahmann, the affective component of the CCAS has been conceptualized as the neuropsychiatry of the cerebellum with five domains relevant for dementia and neurodegenerative disorders: attentional control, emotional control, autism spectrum disorders, psychosis spectrum disorders and social skill set. Behavioral studies in the field of dementia now consider that cerebellar circuits might become a masterpiece of this puzzle. The key role of the cerebellum in the clinical manifestations of dementia must be considered within the framework of the multiple closed loops between the cerebellum, basal ganglia and cerebral cortex, in relation to the predictive nature of the cerebellar computations and rewarding aspects (Olivito, 2020; Iosif et al., 2022). Brain imaging studies have demonstrated a double representation of the sensorimotor cerebellum in the anterior lobe/lobule VIII, a triple cognitive representation in the cerebellar posterior lobe, and a representation in the cerebellum of the intrinsic connectivity networks found in the cerebral hemispheres (Schmahmann, 2022). The universal cerebellar transform reconciles the dual anatomic representation into a unique homeostatic function of the cerebellum, applicable to dementia profiles.

## Author contributions

All authors contributed equally to the conceptualization and management of submitted articles of the Research Topic and to the writing of this editorial.
